# Unaltered NKG2D-CAR T cell function under hypoxia in osteosarcoma *in vitro*

**DOI:** 10.1007/s00262-026-04319-w

**Published:** 2026-02-12

**Authors:** Laura Hidalgo, Patricia Garcia-Rodriguez, Isabel Cubillo, Marta Zubizarreta, Antonio Perez-Martinez, Javier García-Castro

**Affiliations:** 1 1Biomedical Innovation Unit, Centro de Investigaciones Energéticas Medioambientales y Tecnológicas (CIEMAT), 28040 Madrid, Spain; 2 Advanced Therapies Unit., Instituto de Investigación Sanitaria Fundación Jiménez Díaz (IIS-FJD/UAM), 28040 Madrid, Spain; 3 Cellular Biotechnology Unit, Instituto de Salud Carlos III (ISCIII), 28220 Madrid, Spain; 4 Universidad Nacional de Educación a Distancia (UNED), 28015 Madrid, Spain; 5 IdiPAZ-CNIO Pediatric Onco-Hematology Clinical Research Unit, Paediatric Haemato-Oncology Department, School of Medicine, La Paz University Hospital, Universidad Autónoma de Madrid (UAM), CIBERER-, 28046 Madrid, Spain; 6 Instituto de Investigación de Enfermedades Raras (IIER) & Departamento de Desarrollo de Medicamentos de Terapias Avanzadas (DDMTA), Instituto de Salud Carlos III, 28220 Madrid, Spain

**Keywords:** (OS) osteosarcoma, Hypoxia, Immunotherapy, (CAR) chimeric antigen receptor: NKG2D

## Abstract

Cancer is the leading cause of death among children and adolescents in high-income countries. Among solid tumors, osteosarcoma (OS) is the most prevalent primary bone cancer in the pediatric population. For patients who relapse or develop metastases, the survival rate remains at only 30%, with scarce improvement over the past 30 years. Immunotherapy, including chimeric antigen receptor (CAR) T cells, represents a promising approach to treating OS. We observed that despite robust in vitro cytotoxicity of NKG2D-CAR T at low effector-to-target ratios, NKG2D-CAR T cells failed to control tumor growth in our xenograft OS models, highlighting the suppressive tumor microenvironment (TME). In this regard, the hypoxic TME has been widely considered a barrier to effective immunotherapies. Here, we examined the impact of hypoxia on NKG2D-CAR T function in OS in vitro models. We confirmed HIF-1α is highly expressed in our OS models, indicating the potential of hypoxia as a CAR T disruptor. However, hypoxia was not responsible for lowering NKG2D ligands expression for CAR recognition, nor did it impact the expression of major activating or inhibitory immune checkpoints. Crucially, functional assays demonstrated that NKG2D-CAR T cell phenotype, activity, and cytokine secretion remained unaffected and its activity against three-dimensional OS-spheroids was preserved under in vitro hypoxic conditions. Although these findings challenge the idea that hypoxia alone compromises in vitro NKG2D-CAR T efficacy in OS, further studies are needed on how hypoxia interacts with multiple factors of the TME that could modulate CAR T cell behavior.

**Supplementary Information:**

The online version contains supplementary material available at 10.1007/s00262-026-04319-w.

## Background

Osteosarcoma (OS) is the most common bone tumor that affects mainly children and young adults. Current treatments include chemotherapy and surgery, and the 5-year relative survival rate is approximately 70% for patients with localized tumors. However, there have been no remarkable advances in OS therapies over the last three decades and patient outcomes for recurrent and metastatic OS remain poor [1]. Immunotherapies have emerged as promising therapeutic approaches. Monoclonal antibodies against immune checkpoints (ICs) and adoptive T cell therapy, such as CAR T cells, have shown great therapeutic effects in treating melanoma, carcinomas, and hematologic malignancies. However, the results for sarcomas, such as OS, are not satisfactory [2, 3].

The clinical response of solid tumors to CAR T cells is limited [4]. Several obstacles have been described, such as on-target off-tumor toxicity, poor T cell trafficking and persistence, and an immunosuppressive tumor microenvironment (TME). The TME has metabolic and immunological challenges, including the presence of suppressive cytokines and regulatory immune cells, acidic pH, nutrient deprivation, high levels of reactive oxygen species, and hypoxia [5]. Analyzing these hostile conditions individually is key to advancing OS treatment and developing strategies to refine the therapeutic approach.

Hypoxia is a common hallmark of solid tumors, promotes proliferation, metastasis and is associated with treatment failure. Specifically, in OS, it promotes tumor invasion and proliferation [6, 7]. Hypoxia might also generate immunosuppressed TME, upregulating the expression of ICs such as PD-L1 in several types of tumor cells [8] and promoting immune evasion by suppressing MHC-I expression [9]. ICs not only inhibit T cells from patients but also impair the response to therapies based on T cells, including CAR T cells. Since blocking classical ICs, such as the PD-1/PD-1 axis and CTLA-4, is not effective for OS patients [2, 10], we have studied a broad panel of ICs in several OS tumor cell lines with different tumorigenic potentials [11].

The role of hypoxia in T cells is controversial, but several studies have shown that a low O_2_ supply does not directly affect T cell cytolytic capacity [12, 13]. Despite this evidence, hypoxia is considered a major obstacle for CAR T therapy [14–16]. Understanding how therapeutic T cells, such as NKG2D-CAR T cells, perform under these hostile conditions is crucial for optimizing their clinical application. This CAR T therapy can target multiple ligands and has exhibited preclinical efficacy in some solid tumors such as stomach, liver, cervical cancer and OS [17–20].

Our findings suggest that, although the efficacy of NKG2D-CAR T cells in vivo was limited in our OS models, which expressed HIF-1α, hypoxia did not inhibit NKG2D-CAR T cell activity in OS. We demonstrated unchanged NKG2D ligands (NKG2DL) and immune checkpoints (ICs) expression under hypoxia. In addition, the phenotype, cytokine production and killing capacities of NKG2D-CAR T cells against OS cells were unaltered in both the OS-monolayer and OS-spheroid in hypoxic conditions. These data together should be taken into consideration when evaluating hypoxia as a main barrier to CAR T therapy.

## Materials and methods

### OS cell lines culture

The human OS cell lines 143B, MNNG/HOS, Saos-2, and U-2 OS were cultured in DMEM (Lonza) supplemented with 10% fetal bovine serum, 100 IU/ml penicillin/streptomycin (Lonza), and glutamine (2 mM) (complete medium). OS cell lines were regularly tested for Mycoplasma detection (Lonza). For authentication, human OS cells were examined via STR analysis (IIBM, Madrid).

For in vitro assays, OS cell lines were transduced with lentiviral vectors to express GFP, using PL-SIN-EF1α-EGFP plasmid from Addgene (Ref: 21,320), or with GFP and Luciferase using pHRSIN-SFFV-LUC-IRES-GFP plasmid kindly provided by Dr. Antonio Rodríguez (Autonomous University of Madrid).

Cells were maintained in common incubators with an atmospheric oxygen concentration (21% O_2_) and 5% CO_2,_ also referred to as normoxia or normoxic conditions throughout the manuscript. For hypoxia, cells were cultured inside a hypoxic chamber for cell culture (BioSpherix), with an O_2_ and CO_2_ controller. Hypoxic conditions were set at 1% O_2_ and 5% CO_2_.

To establish the spheroid model, 2 × 10^4^ OS cells were seeded with complete medium in P96-well low fixation Nunclon Sphera U-bottom microplates (Thermo Fisher) followed by 10 min of centrifugation at 200 g. Cells were self-assembled into spheroids after 48 h of culture in a normoxic or hypoxic environment. The spheroids were cocultured with 4 × 10^4^ NKG2D-CAR T cells. OS live cells were analyzed by flow cytometry after 24 h of coculture. Images were acquired by fluorescence microscopy (Leica DM IL LED) and analyzed by ImageJ software.

### HIF-1α expression

OS cells were seeded at 15 × 10^3^ cells/well in P12 chambers (Ibidi) under normoxic and hypoxic conditions for 48 h. The slides were fixed, permeabilized, and blocked. Cells were stained with a monoclonal HIF-1α antibody (Clone 241,809, R&D Systems) at 1:200, followed by secondary antibody anti-mouse IgG (H + L)-Alexa Fluor 488 (AB_2536161, Thermo Fisher) at a ratio of 1:2000 and mounted with ProLong Diamond mountant with DAPI (Thermo Fisher). Images were acquired on the STELLARIS Confocal microscope (Leica) at 20x. HIF-1α expression was quantified using ImageJ software. HIF-1α expression was calculated as the mean fluorescence intensity under hypoxia divided by the mean fluorescence intensity under normoxia. Immunohistochemistry was applied to formalin-fixed paraffin sections of 5 µm thick 143B and MNNG/HOS xenograft tumors (*n* > 3). The HIF-1α antibody was used at 2.5 µg/µl on BOND-MAX (Leica). Staining was performed according to the manufacturer’s staining protocol. The sections were counterstained with hematoxylin and acquired in Nanozoomer (Hamamatsu). Protein expression was scored in QuPath software.

### NKG2D-CAR T transduction

Leukocyte Reduction System cones from healthy donors were obtained from the Biobank Hospital Universitario Puerta de Hierro Majadahonda (HUPHM)/Instituto de Investigación Sanitaria Puerta de Hierro–Segovia de Arana (IDIPHISA) (PT17/0015/0020 in the Spanish National Biobanks Network). They were processed under standard operating procedures with the appropriate approval of the Ethics and Scientific Committees. PBMCs and T cells were isolated following the protocol described in previous studies [21].

The NKG2D-41BB-CD3z construct was kindly provided by Dr. Lucia Fernandez at Spanish National Cancer Research Centre (CNIO), previously described [20, 22]. Lentiviral vectors were produced by the Vector Viral Unit of the Spanish National Center for Cardiovascular Research (CNIC).

Purified T cells were activated using Dynabeads Human T-Activator CD3/CD28 (Gibco) in X-VIVO 15 media (Lonza) supplemented with IL-2 (Miltenyi Biotec). The next day, they were transduced with lentiviral particles at MOI 2. CAR T cells were maintained at 10^6^ cells/ml. Beads were removed on day 7. Experiments were performed with resting CAR T cells from day 10 after transduction. The transduction efficacy was always near 100%.

### Cell-mediated cytotoxicity in vitro assay

Limiting dilution: 143B and MNNG/HOS cells were seeded in 6-well plates at 1 × 10^5^ cells per well. After 24 h, OS cells were cocultured with NKG2D-CAR T cells at effector-to-target (E/T) ratios of 1:10, 1:100, and 1:1000. The cocultures were maintained for up to 23 days. Cytotoxic activity was assessed by flow cytometry. The percentage of viable tumor cells was based on 7-AAD-negative staining.

Hypoxia: luciferase-expressing OS cell lines were seeded at 7 × 10^3^ cells per well into 96-well plates. OS cells were cultured overnight in normoxic or hypoxic conditions. NKG2D-CAR T cells were added at E/T ratio of 1:5 and 1:1 in 200 μl of X-VIVO 15.

(i) To test OS cell viability under hypoxia, IVISbrite D-Luciferin Potassium Salt (Revvity) was added to the wells at a final concentration of 2 mM. Luminescence readouts were performed at indicated times after the addition of NKG2D-CAR T cells using the Infinite M200 plate reader (Tecan). The mean ± SD of the luminescence values of OS + NKG2D-CAR T cells (from three healthy donors) relative to cells without NKG2D-CAR T cells (CTRL OS) at each time point was calculated.
(ii) For testing cytokine production, supernatants from cocultures were collected at 48 h and IFN-γ, TNF-α, IL-2, and MICA levels were tested by ELISA (BioLegend and R&D Systems). The mean ± SD of cytokine production (from 3–6 donors) was calculated.

### Flow cytometry

OS cell lines and CAR T cells were stained with combinations of fluorochrome-labeled anti-human antibodies against CD276 (B7-H3)-FITC (Clone REA1094; Miltenyi Biotec), CD73-APC (Clone AD2, Biolegend), CD274 (PD-L1)-FITC (Clone MIH2, Biolegend), HMGB1/HMG-1-PE (Clone IC1690P, R&D Systems), Galectin-9-APC (Clone 9M1-3, Biolegend), CD270 (HVEM)-APC (Clone 122, Biolegend), CD40-FITC (Clone 5C3, Biolegend), CD252 (OX40L)-PE (Clone 11C3.1, Biolegend), CD137L (4-1BB Ligand)-APC (Clone 5F4, Biolegend), MICA-APC (Clone 159,227, R&D Systems), MICB- APC (Clone 236,511, R&D Systems), UBLP-1- Alexa Fluor 488 (Clone 170,818, R&D Systems), ULBP-2/5/6-PE (Clone 165,903, R&D Systems), ULBP-3-PE (Clone 166,510, R&D Systems), ULBP-4-APC (Clone 709,116, R&D Systems), NKG2D-PEVio770 (Clone REA797, Miltenyi Biotec), CD25-PEVio770 (Clone REA945, Miltenyi Biotec), PD1-PEVio770 (Clone REA1165, Miltenyi Biotec), NTBA-PE (Clone NT7, Biolegend), TIM-3-FITC (Clone F38-2E2, Biolegend), CD107a-PE (Clone H4A3, Biolegend), CD69-PECy7 (Clone FN50, Biolegend). Intracellular staining was performed using GranzB-Pacific Blue (Clone GB11, Biolegend) and Fixation and Permeabilization Buffer Kit I (R&D system). Live mitochondrial membrane potential was detected by Image-iT TMRM Reagent ( Invitrogen). The data were analyzed with Quant Analyzer 10 (Miltenyi Biotech) and FlowJo software v10.9.0.

### In vivo studies

OS xenografts were established by subcutaneous injection of 1 × 10^6^ 143B and MNNG/HOS cells into the right flank of 8–12-week-old NOD.Cg-Prkdc^scid Il2rg^tm1Wjl/SzJ (NSG) mice (Jackson Laboratory). All animal procedures were performed at Instituto de Salud Carlos III (Madrid, Spain) in accordance with institutional and regional ethical guidelines. This study was approved by Consejería del Medio Ambiente de la Comunidad de Madrid (PROEX 133.7/21).

The mice received a single intravenous dose of 5 × 10^6^ ex vivo expanded NKG2D-CAR T cells. Tumor dimensions—length (L), width (W), and height (H)—were measured at regular intervals with calipers. The tumor volume was calculated using the formula $V=[\mathrm{L}\times \mathrm{W}\times \mathrm{H}]/2$. The area under the curve was calculated with GraphPad Prism. Mice were euthanized under ethical endpoints (< 1500mm^3^).

### Statistical analysis

GraphPad Prism 9.1.1 software was used for statistical analysis. The statistical tests used are indicated in the figure legends. *p* values of less than 0.05 were considered significant.

## Results

### NKG2D-CAR T cells exhibit effective in vitro cytotoxicity but fail to control tumor growth in vivo

**Fig. 1:**
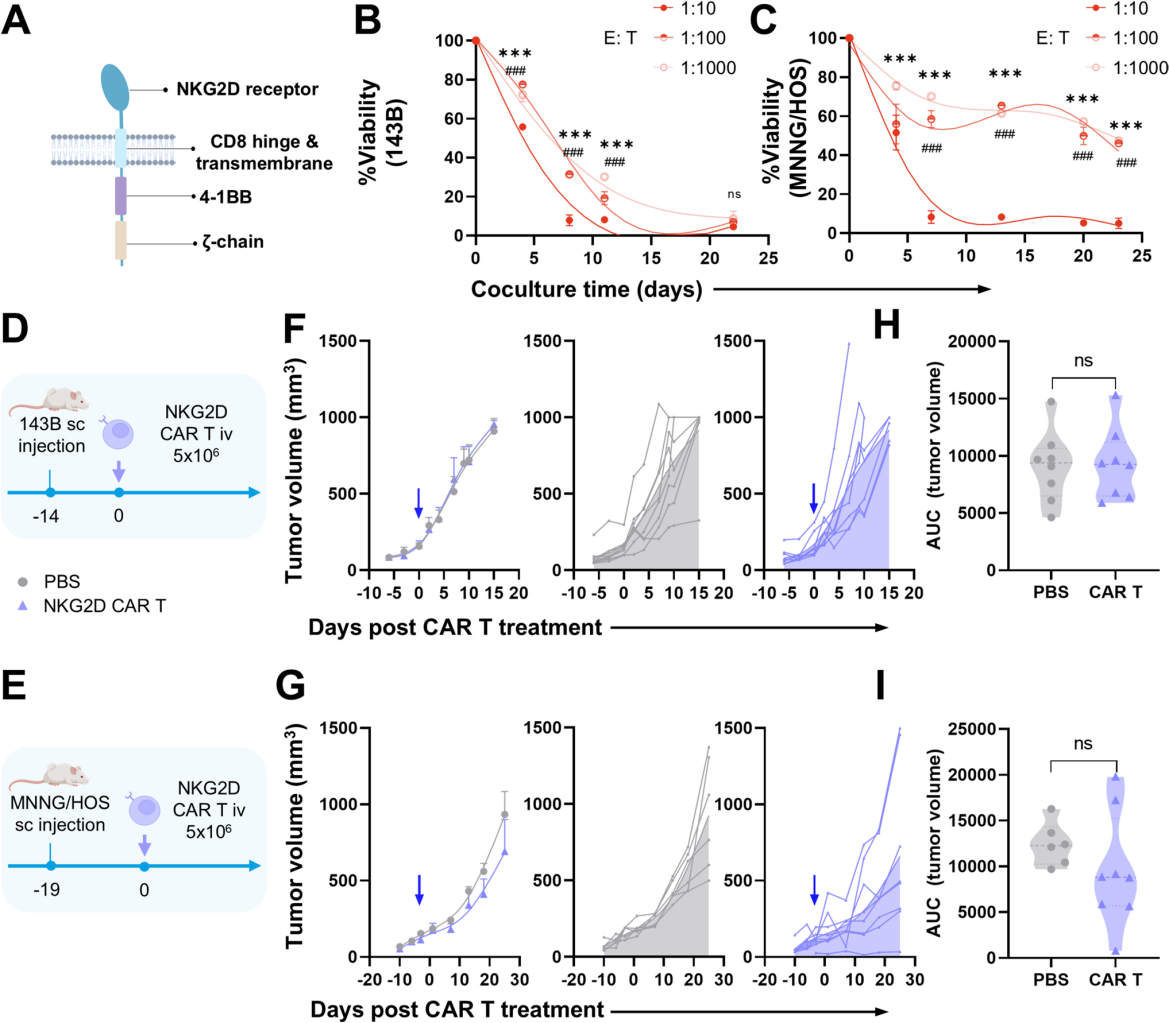
Potent in vitro cytotoxicity of NKG2D-CAR T contrast with poor in vivo efficacy. **A** Schematic illustration of NKG2D-CAR construct domains. **B** Viability percentage of 143B and **C** MNNG/HOS cocultured with NKG2D-CAR T cells at low effector/target (E/T) ratios at different timepoints. Viability was measured by 7-AAD^−^ OS cells by flow cytometry. Data represent the mean ± SD, ***p < 0.001 (E/T; 1:10 vs 1:1000), ### < 0.001 (1:10 vs 1:100) by Tukey’s test. **D** Schematic illustration of in vivo xenograft models 143B and **E** MNNG/HOS grafted subcutaneously (sc) and treated with NKG2D-CAR T intravenously (iv). **F** Tumor volume of 143B-bearing and **G** MNNG/HOS- bearing mice after treatment. Tumor volume was measured by a caliper. Data represent the mean ± SEM. (**H** and **I**) The area under the curve (AUC) was calculated to statistically compare antitumoral control of treatment overtime. Not-statistically (ns) differences were observed by t test

To assess the specificity and cytotoxic capacity of NKG2D-CAR T cells against OS (Fig. 1A), we performed in vitro cocultures with OS cell lines (143B and MNNG/HOS) at low E/T ratios. Notably, NKG2D-CAR T cells efficiently eliminated 143B cells even at a 1:1000 ratio, indicating robust antitumor activity under strict conditions (Fig. 1B). MNNG/HOS cells showed significant partial resistance to CAR T at very low ratios (Fig. 1C).

We developed two xenograft models to test CAR T cells with these aggressive OS cell lines (Fig. 1D, E). However, compared with PBS-treated controls, NKG2D-CAR T cells did not demonstrate tumor control (Fig. 1F, G). Analysis of tumor growth, assessed by the area under the curve (AUC) of tumor volume over time, revealed not significant differences between PBS-treated and CAR T-treated mice (Fig. 1H, I).

### OS xenograft tumors expressed HIF-1α

**Fig. 2:**
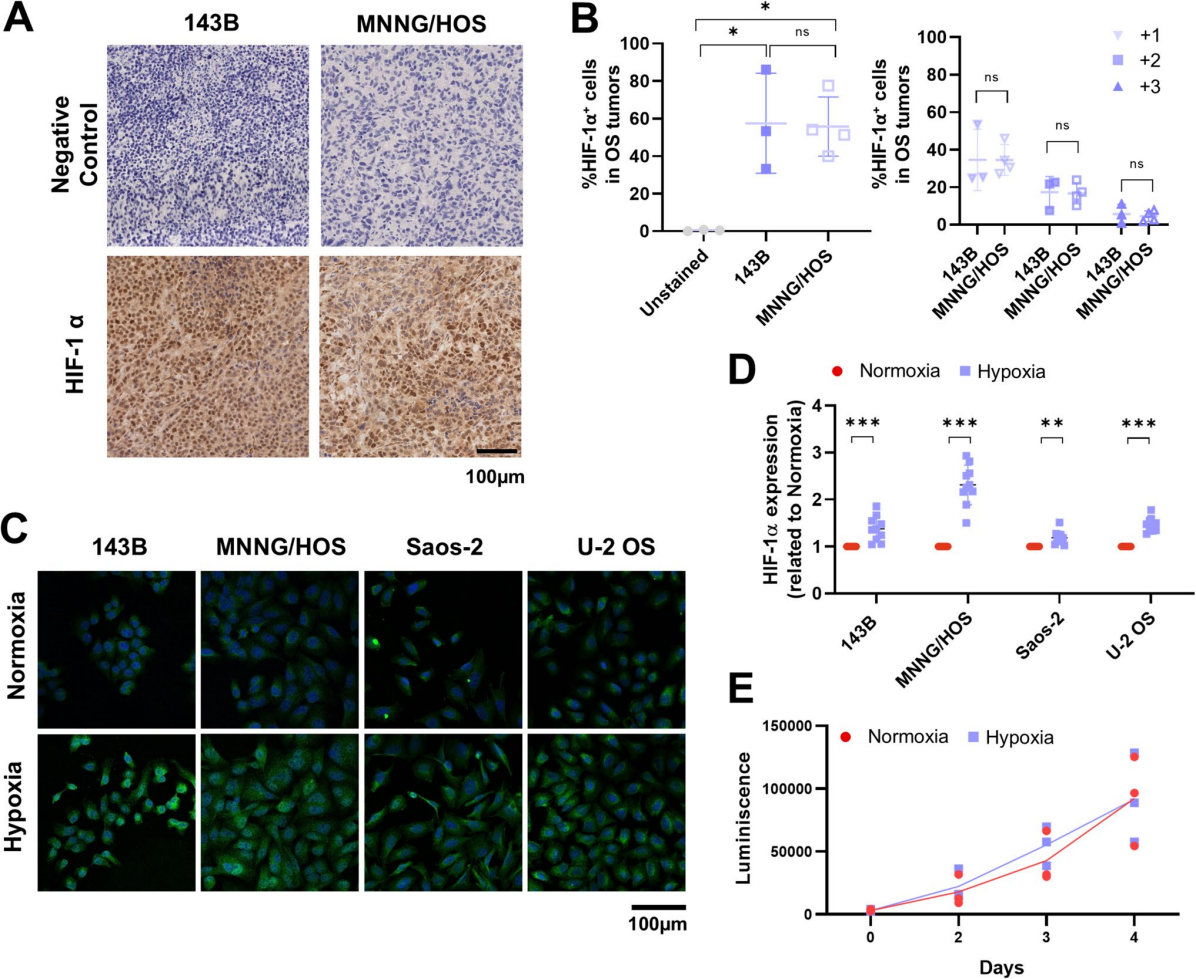
Regulation of HIF-1α expression in vivo and under hypoxia. **A** Immunohistology slides stained with HIF-1α (brown) and counterstained with hematoxylin (purple). The scale bar represents 100 µm. **B** Quantified the percentage of HIF-1α^+^ cells by QuPath. DAB intensity thresholds were established as > 0.2 (+ 1), > 0.4 (+ 2) and > 0.6 (+ 3) of at least 3 independent samples. **C** Immunofluorescence (IF) staining of HIF-1α on human OS cells after 48 h of culture under normoxic (21% O_2_) and hypoxic (1% O_2_) conditions. OS cells were incubated with the anti-HIF-1α antibody followed by an Alexa-488-conjugated secondary antibody (green). DNA was stained with DAPI (blue). Images were captured by confocal microscopy at 20x, and the scale bar represents 100 µm. **D** HIF-1α expression was quantified as the mean fluorescence intensity divided by the mean fluorescence intensity in the control condition (normoxia). Data represent the mean ± SD of two independent experiments (9 pictures analyzed/well/experiment). ns = not significant, **p < 0.01, ***p < 0.001 by unpaired t test. (**E**) Live 143B-OS cells were determined by luminescence measurements. It is represented as mean ± SD of 3 independent experiments

Given the discrepancy between in vitro efficacy and the lack of in vivo tumor control, we hypothesized that tumor-intrinsic factors within the microenvironment, such as hypoxia, might limit CAR T cell function. Therefore, we analyzed the expression of HIF-1α in OS tumor sections. HIF-1α was significantly expressed, suggesting the presence of a hypoxic TME in both OS models [6, 7] (Fig. 2A, B). To analyze this factor in vitro, OS cell lines were cultured under low-oxygen conditions, which successfully induced HIF-1α expression (Fig. 2C, D) without affecting viability (Fig. 2E). Therefore, this in vitro model has allowed us to study NKG2D-CAR T-OS interactions under hypoxic stress.

### Hypoxia does not affect NKG2D ligands expression in OS cell lines in vitro

In humans, the NKG2D receptor recognizes several ligands, including the MHC class I-related chain molecules A and B (MICA and MICB) and the UL16-binding proteins (ULBP). Tumor cells can cleave these NKG2DL as a mechanism of antigen escape. To investigate this mechanism, we examined the expression of these ligands in vivo and in vitro.

**Fig. 3:**
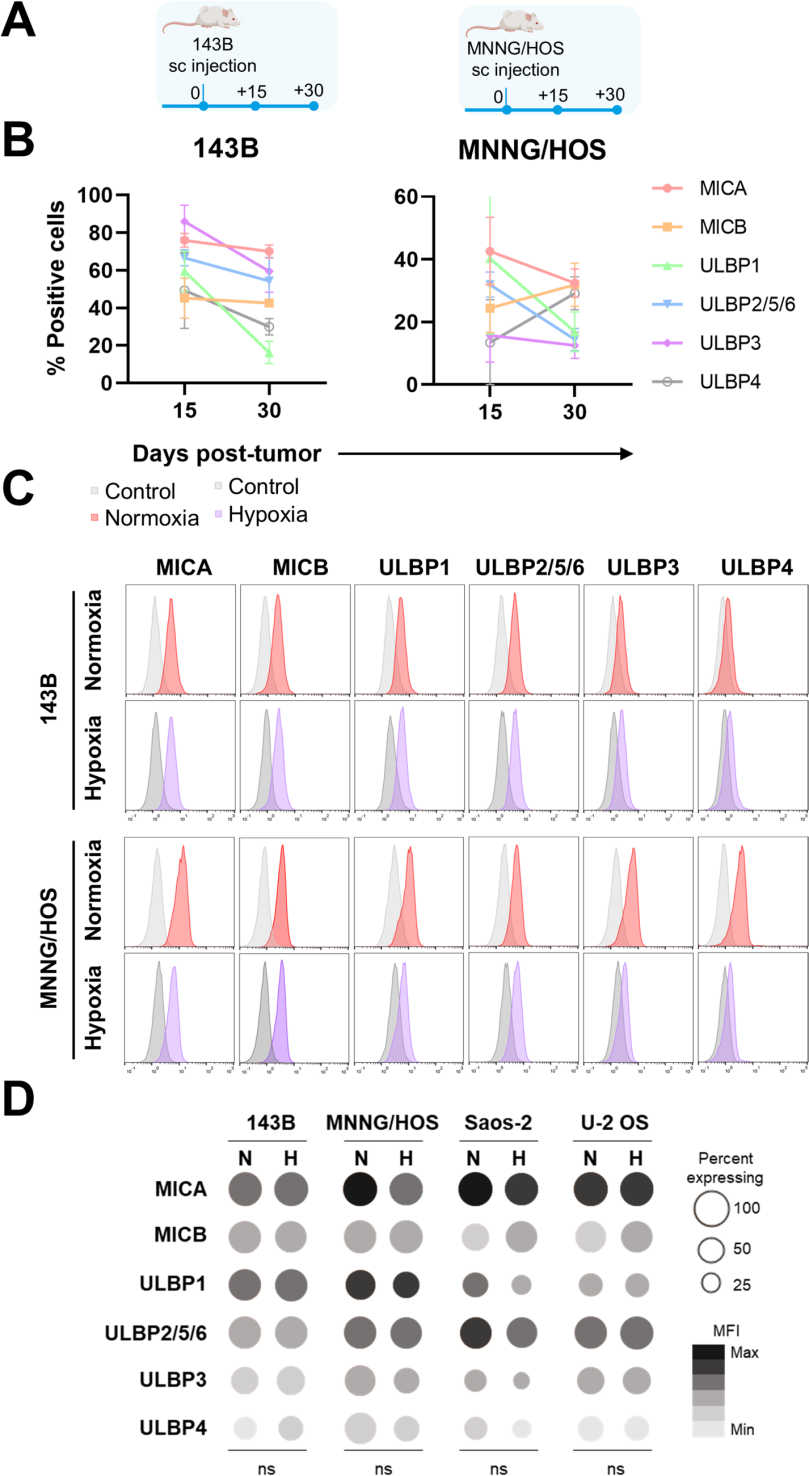
Regulation of NKG2DL expression in murine OS samples and human OS cell lines at low O_2_ levels. Analysis of MIC and ULBP ligands expression in OS measured ex vivo and in vitro by flow cytometry. **A** Schematic illustration of in vivo xenograft models. **B** Percentage of NKG2DL in ex vivo xenograft OS cells 15 and 30 days after engraftment. **C** Representative histograms from two independent experiments of NKG2DL expression are shown 48 h after seeding. Red—expression on normoxia; purple—expression on hypoxia; gray—negative control. **D** The diagram shows the mean of the percentage of positive cells (dot size) and the MFI (gray gradient) for each marker in each cell line and condition (*N* = Normoxia; *H* = Hypoxia). ns = not significant differences according to two-way ANOVA with Sidak post hoc test

We first examined the expression of NKG2DL in OS cells retrieved from two in vivo xenograft models at different timepoints (Fig. 3A). We observed downregulation of some ligands in both models during tumor development (Fig. 3B). However, antigen loss was incomplete, suggesting that impaired CAR T efficacy was not due primarily to a loss of target recognition. Additionally, to assess whether the expression of these ligands is regulated by hypoxia, we cultured OS cell lines under either 21% O_2_ (normoxic) or 1% O_2_ (hypoxic) conditions for 48 h. We observed a slight reduction in the mean fluorescence intensity (MFI) of MICA and in the percentage of some ULBPs in MNNG/HOS and Saos-2 OS cells cultured under hypoxic conditions (purple histograms) for 48 h, compared with those cultured under normoxic conditions (red histograms) (Fig. 3C, D and Suppl. Figure 1). These differences were not statistically significant (Fig. 3D).

### Immune checkpoint expression in OS is independent of hypoxic regulation

ICs play a key role in regulating the T cell response, which also affects CAR T cell efficacy. The expression of these molecules can be regulated by multiple factors such as hypoxia, often in a HIF-1α-dependent manner [8]. To better understand the immunosuppressive landscape of OS and how hypoxia may influence immune evasion, we characterized the expression of a broad panel of inhibitory and stimulatory ICs on four human OS cell lines cultured under hypoxic conditions.

**Fig. 4:**
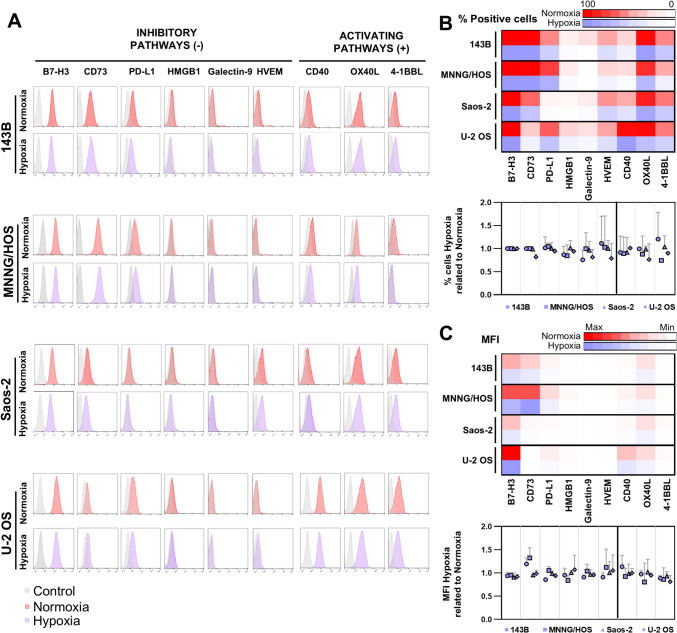
Expression of IC on OS cell lines in normoxia and hypoxia. The expression of inhibitory and activating ligands was measured by flow cytometry on OS cell lines cultured in normoxia and hypoxia for 48 h. **A** Representative histograms of ICs expression are shown. Red—expression on normoxia; purple—expression on hypoxia; gray—negative control. The percentage of positive cells and MFI of each IC were analyzed and the mean of both parameters of three independent experiments was calculated as shown in the right panels. **B** Up: Heatmap of the mean of positive cells percentage for each IC (columns); Red—normoxia, purple—hypoxia (lines). The color in the heatmap varies from white for lower expression to red/purple for higher expression as shown in the color bar above. Down: Percentage of positive cells of each IC on hypoxia related to normoxia of each experiment was calculated (ratio). Graph represents the mean of the ratios calculated. **C** Up: Heatmap of the mean of MFIs for each IC (columns); Red—normoxia, purple—hypoxia (lines). The color in the heatmap varies from white for lower MFI to red/purple for higher MFI as shown in the color bar above. Down: MFI of each IC on hypoxia related to normoxia of each experiment was calculated (ratio). Graph represents the mean of the ratios calculated

All OS cell lines studied expressed high levels of B7-H3 under normoxia (red histograms), as we have previously shown [21]. B7-H3 was the inhibitory IC with the highest expression in our cells followed by CD73, with a more variable expression (Fig. 4A, B, C). Both 143B and MNNG/HOS cell lines expressed high levels of CD73, while the expression was lower in Saos-2 and almost negative in U-2 OS cells (Fig. 4A, B, C). OS cell lines showed highly variable expression of PD-L1, independent of the degree of aggressiveness of the OS cell line. In addition, we analyzed the expression of other inhibitory molecules, such as HMGB1, galectin-9, and HVEM. None of these proteins were expressed in the OS cell lines studied (Fig. 4A, B, C).

With respect to stimulatory pathways, OS cells mostly expressed OX40L and to a lesser extent 4-1BBL. However, CD40 was not expressed in most of the OS cell lines apart from U-2 OS (Fig. 4A, B, C).

To analyze whether the expression of these ligands was regulated by hypoxia, we cultured OS cell lines under either normoxic or hypoxic conditions for 48 h. Next, we studied the expression of inhibitory and activating ICs. Our results indicated that low concentrations of oxygen (purple histograms) did not affect the expression of any of the ICs studied.

### NKG2D-CAR T function is not altered under hypoxic conditions

**Fig. 5:**
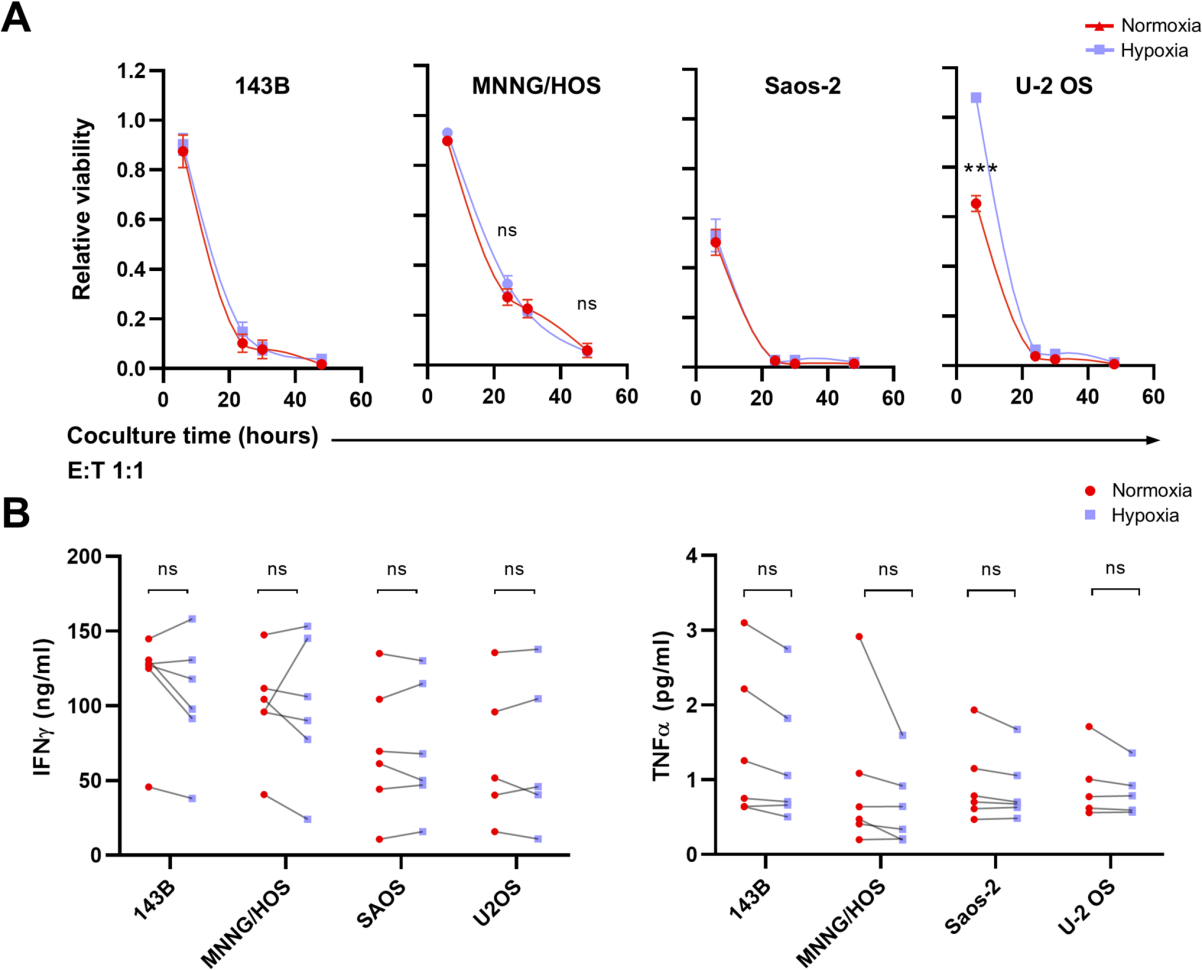
In vitro antitumor activity of NKG2D-CAR T cells under hypoxia. Luciferase-expressing OS tumor cells are cocultured with NKG2D-CAR T cells at 1:1 effector/target cells ratio in normoxic vs hypoxic conditions. **A** Live OS cells were determined by luminescence measurements after NKG2D-CAR T cell coculture. Data were normalized to OS cells without CAR T cells (OS cell control). It is represented as mean ± SD of experiments with CAR T cells from 6 different donors. ***, *p* < 0.001 by two-way ANOVA with Tukey´s post hoc test. **B** IFN-γ and TNF-α levels are quantified by ELISA in the cocultured supernatants after 48 h

NKG2D-CAR T cells show in vitro antitumor effects on OS cell lines [20]. However, there are no data about their function under low concentrations of O_2_. To answer this question, we cocultured NKG2D-CAR T cells and OS cells under normoxic and hypoxic conditions and evaluated the cytotoxic activity of NKG2D-CAR T cells at different E/T ratios. To that end, we analyzed the viability of OS cells after coculture at different timepoints. We employed a luciferase-based assay where viability is measured by bioluminescence intensity since it is proportional to the number of viable luciferase-expressing target cells. We observed that NKG2D-CAR T cells exhibited significant cytotoxicity against OS cell lines under both conditions (Fig. 5A) even at low ratios (Suppl. Figure 2A).

**Fig. 6:**
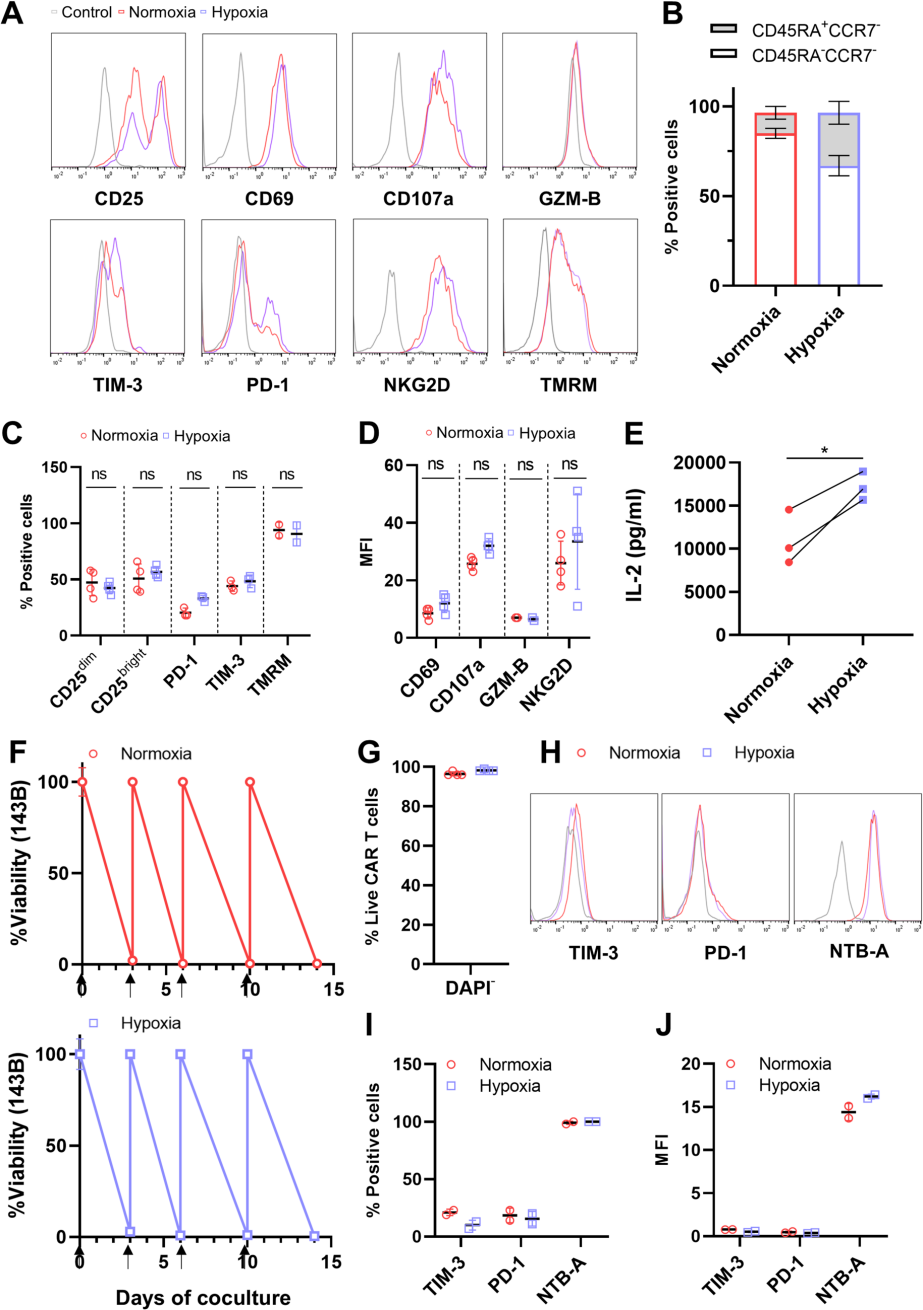
NKG2D-CAR T phenotype after coculture with 143B-OS. The expression of several CAR T cell markers was measured by flow cytometry on live NKG2D-CAR T cells (CD45^+^ DAPI^−^) cocultured with 143B OS cells under normoxia (red) and hypoxia (purple) in 6-well plates. Each dot represents a different donor. Panels A to E show data after 48 h of coculture. **A** Representative histograms of the indicated markers. **B** Percentage of CAR T cell populations based on CCR7 and CD45RA expression. **C** The percentage of positive cells and **D** mean fluorescence intensity (MFI) of the markers were analyzed, and the mean of both parameters was calculated as shown in the graphs. **E** IL-2 levels were quantified by ELISA in the cocultured supernatants. Panels F to J show data from the repetitive tumor challenge assay. NKG2D-CAR T cells were exposed to four rounds of coculture with 143B OS cells at a 1:1 ratio under normoxia (red) and hypoxia (purple) in 96-well plates. **F** Live 143B OS cells were quantified by luminescence measurements. **G** Percentage of live CAR T cells at the end of the assay (14 days). **H** Representative histograms of exhaustion markers. The percentage of positive cells **I** and MFI **J** of these markers was analyzed, and the mean of both parameters was calculated as shown in the graphs

We also compared the phenotype of NKG2D-CAR T cocultured with 143B OS cells under normoxia and hypoxia. The expression of different activation (CD25, CD69), function (CD107a, GZM-B, CCR7, CD45RA, NKG2D), and exhaustion markers (TIM-3 and PD-1) is similar under both conditions (Fig. 6A-D). To indicate the metabolic health of the cells, we examined the mitochondrial membrane potential using TMRM staining. No differences were observed between CAR T cells under normoxia and hypoxia (Fig. 6A, D).

In addition, to evaluate the recursive killing potential of CAR T cells, we also established an in vitro tumor rechallenge model. We analyzed cytotoxicity (Fig. 6F) and after four rounds of tumor exposure, viability and exhaustion markers in CAR T cells, finding that hypoxia did not alter any of the parameters (Fig. 6G-J).

Another important characteristic of CAR T cell activation is their ability to secrete proinflammatory cytokines. Therefore, we collected the supernatants from the cocultures in both conditions and analyzed the secretion of IFN-γ, TNF-α, and IL-2 by ELISA. The levels of IFN-γ and TNF-α remained comparable (Fig. 5B and Suppl. Figure 2B), while IL-2 was higher under hypoxia (Fig. 6E).

On the other hand, we studied the expression of NKG2DL on tumor cells after coculture. We observed a reduction in the expression of MICA/B and an increase in soluble MICA in both coculture conditions compared to tumor cells alone (Suppl Fig. 3). However, the concentration of soluble MICA was significantly higher in normoxia compared to hypoxia (Suppl Fig. 3B). ULBPs expression remained unaltered (Suppl Fig. 3A).

### NKG2D CAR T cells effectively target OS-spheroids under hypoxia

To accurately model the structural characteristics of solid tumors, we developed 3D OS-spheroids to assess the cytotoxic potential of NKG2D-CAR T cells. We used all OS cell lines expressing GFP (GFP-OS) in ultralow-adherence plates under normoxic and hypoxic conditions. After 48 h of seeding, we compared their size, form, and GFP fluorescence intensity by imageJ software, which revealed similar characteristics in both conditions (Suppl. Figure 4A and B).

**Fig. 7:**
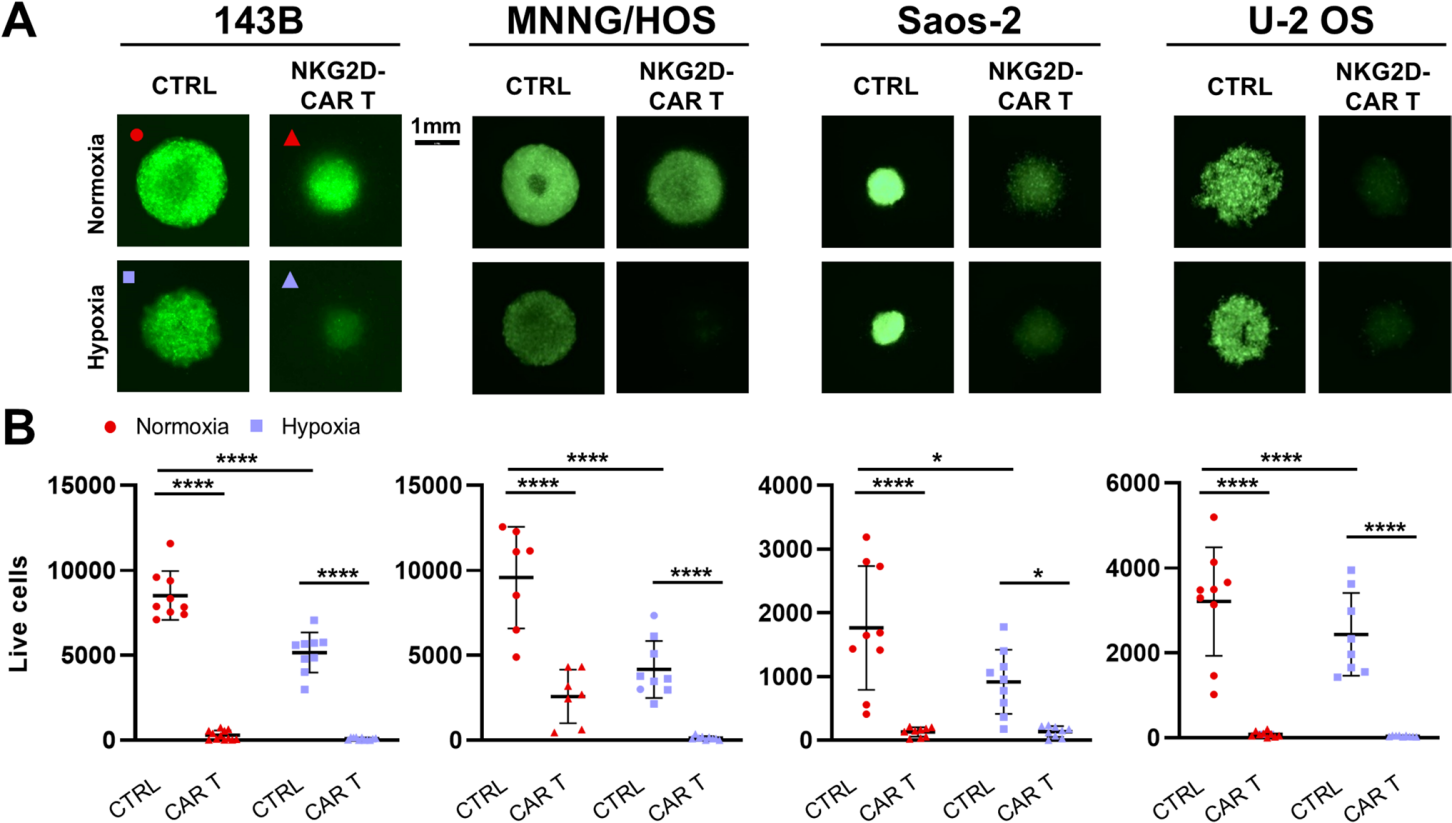
NKG2D-CAR T cells efficacy under 3D and hypoxic environment. GFP-OS cells spheroids were cultured under normoxic or hypoxic conditions for 48 h. Then, NKG2D-CAR T cells were added and they were cocultured for 24 h under indicated conditions. **A** Representative fluorescence images (4x) of GFP-OS cell lines after 24 h of coculture with NKG2D-CAR T cells. **B** OS viable cells were determined by flow cytometry as GFP^+^7AAD^−^. Data shown are mean ± SD of three independent experiments from 3 different donors (*n* = 3). All experiments were performed in triplicate. *, *p* < 0.05; **, *p* < 0.01; ***, *p* < 0.001; ****, *p* < 0.0001 by two-way ANOVA with Tukey´s post hoc test

Once OS-spheroids were established, we added NKG2D-CAR T cells from different donors and checked their activity after 24 h of coculture. The fluorescence images revealed that NKG2D-CAR T cells reduced the size of the OS-spheroids (Fig. 7A). The quantification of the number of live OS cells by flow cytometry also revealed that the NKG2D-CAR T cells killed OS cells even in 3D cultures (Fig. 7B). Moreover, as previously shown in the 2D models, the cytotoxicity of NKG2D-CAR T cells was similar between normoxia (red triangles) and hypoxia (purple triangles) (Fig. 7B). Overall, these data suggest that other tumor-intrinsic factors beyond hypoxia or microenvironmental factors may contribute to immune resistance in OS.

## Discussion

CAR T cell therapy has emerged as an alternative for treating cancer, but limited clinical benefit has been demonstrated in the treatment of solid tumors because of multiple barriers within the TME. Our in vitro data indicate that NKG2D-CAR T cells exhibit potent and specific cytotoxicity against OS cell lines, including suboptimal E/T ratios, compared with other CAR T cells, which exhibit ratio-limited potency [23]. Despite this strong in vitro cytotoxicity, NKG2D-CAR T cells failed to control tumor growth in our xenograft OS mouse models.

In this context, early-phase CAR T clinical trials targeting antigens such as HER2 and B7-H3 in OS have also demonstrated limited clinical benefit, with most patients achieving only transient disease stabilization (stable disease in 4 of 16 and 1 of 16 patients, respectively) before progression [24, 25]. Moreover, OS patients showed better in vivo expansion of GD2-CAR T, but this expansion was not enough to achieve positive responses in this clinical trial [26]. We are currently evaluating the safety of allogeneic memory NKG2D-CAR T cells for treating pediatric patients with advanced sarcomas in a phase I clinical trial (NCT06087341).

Preclinical studies evaluating CAR T therapies in OS models have shown contrasting efficacies depending on the experimental design, but most of them have low therapeutic effects [27, 28]. Frequently optimized conditions are used, including CAR T administration with low tumor burdens, repeated CAR T cell dosing regimens, intratumoral injection, or combination strategies [29, 30]. Our in vivo models have more stringent settings, to mimic the clinical outcome. Specifically, tumors are visible and established when CAR T cells are injected, which results in a lower antitumoral response than previously published data [20]. This discrepancy underscores the complexity of TME, which imposes significant suppressive pressures on immune effector cells.

Hypoxia is often described as a major barrier in this immunosuppressive TME. In OS, it promotes malignant phenotypes and a high risk of metastasis [31]. Hypoxia and immune status are associated with the prognosis of OS patients [32, 33]. These hypoxia-induced alterations can confer resistance to conventional therapies, such as chemotherapy, radiotherapy, and immunotherapy [32, 34]. This premise is also assumed in CAR T cell therapy, although very few studies address this issue. Hypoxia might be particularly relevant to NKG2D-CAR T therapy since it has been described as the downregulation of the NKG2DL, MICA, under low O_2_ concentrations, affecting NK cell activity [35]. Given the histological presence of HIF-1α in the tumor sections analyzed, we investigated hypoxia as a potential contributor to the lack of efficacy of NKG2D-CAR T cells.

We analyzed changes in the expression of NKG2DL during tumor development. Although most of the tumor cells maintained the expression of these ligands, we observed a downregulation ex vivo in some of the OS cell lines studied. The regulation of MICA expression comprises complex mechanisms. Our in vitro results showed that hypoxia did not affect the expression. However, following coculture with NKG2D-CAR T cells, tumor cells downregulated MICA expression on the membrane and increased the soluble ligand in the supernatant. Notably, the expression of other ligands was unaffected by any of the conditions.

We also explored the expression of IC molecules in OS cell lines. One of the best-known inhibitors is the PD-1/PD-1 axis. In OS, high expression of PD-L1 is related to metastasis and poor prognosis [36]. However, the clinical results in which the PD-1/PD-L1 pathway is blocked are disappointing [2, 10]. Therefore, there is an urgent need to explore new pathways in OS. For that, we evaluated the expression of several ICs not only for inhibitory pathways but also for activating pathways in four human OS cell lines with different phenotypes and tumorigenic capacities [11]. B7-H3 and CD73 are highly expressed in OS cells. The inhibition of these pathways has emerged as a promising strategy for tumor treatment. Several laboratories, including ours [21], have demonstrated that CAR T cells targeting B7-H3 are effective against OS tumors [37]. In addition, our results indicate that the highly aggressive OS cell line, MNNG/HOS has the most immunosuppressive phenotype among the OS cell lines studied. This might be the cause of lower NKG2D-CAR T efficacy compared to 143B at limiting E/T ratios. The expression of these ICs in other solid tumors can be regulated by modulatory factors such as hypoxia in a HIF-dependent manner [8], but data concerning OS are lacking. Our findings suggest that although HIF-1α is upregulated, hypoxia did not significantly affect the expression of the ICs studied in our OS model.

To gain deeper insight into potential CAR T cell regulatory mechanisms, we assessed whether a low O_2_ concentration might be an obstacle to NKG2D-CAR T functionality. Notably, hypoxia does not affect the effectiveness of NKG2D-CAR T cells in vitro, neither in short-term coculture nor after four rounds of tumor exposure. Our results show that hypoxia does not impair the phenotype, cytotoxic capacity, or production of proinflammatory cytokines by NKG2D-CAR T cells against OS cell lines. We only observed changes in IL-2, with higher production under hypoxic conditions. A similar increase in IL-2 secretion has been observed when T cells are activated under low-oxygen conditions compared with activation at 21% O_2_ [38].

Given that monolayer cultures do not represent the complexity of the TME, we also developed 3D OS-spheroids under non-adherent conditions. These 3D cultures represent interesting tools for analyzing immune-tumor cell interactions without having to resort to in vivo models. Specifically, we used them to evaluate the killing capacity of NKG2D-CAR T cells in OS models that mimic human tumors [39]. It has been proposed that there is a delay in tumor cell lysis in spheroids compared with monolayer cocultures [40]. However, we have shown that NKG2D-CAR T cells kill tumor cells and disrupt their three-dimensional structure. Although we have not directly compared 2D and 3D models, we rarely obtained live GFP-OS cells after 24 h of coculture with NKG2D-CAR T cells, suggesting the in vitro efficacy of our therapy even in more resistant models.

Oxygen gradients can be engineered in 3D models for difficult immune cell infiltration [40]. To investigate whether this could be a barrier in our system, we also developed OS-spheroids under hypoxic conditions, such as those developed under normoxia. We obtained results identical to those of 2D, with no impact of oxygen deprivation on NKG2D-CAR T cell function.

These results are consistent with those reported in the few studies that have addressed the functional consequences of hypoxic conditions in CAR T cells. One of the first works related to this topic suggested that hypoxia affects CD19  and BCMA-specific CAR T cell expansion, differentiation, and cytokine production [41]. Nevertheless, in this study, both CAR T cell types exhibited similar cytotoxic activity compared with normoxic and hypoxic conditions. Similar effects have been obtained in hypoxia-responsive CAR T cells against solid tumors, where the killing capacity of these CAR T cells was not affected by low levels of O_2_ [42–44].

Finally, our data suggest that other tumor-intrinsic factors may be responsible for the lack of efficacy of antitumoral NKG2D-CAR T therapy. We have studied the isolated effect of hypoxia in our model, but other factors, such as nutrient limitation, may also impact T cell metabolism and function and need to be considered [45–47]. In addition, rather than acting directly on CAR T cells, hypoxic conditions may reshape the tumor immune landscape by promoting the polarization of myeloid cells to an immunosuppressive state [48]. In parallel, hypoxic tumors upregulate the secretion of chemokines, which actively recruit immunosuppressive cell populations, including regulatory T cells and myeloid-derived suppressor cells [49]. These effects create a TME that can affect CAR T cell expansion, persistence, and cytotoxicity, ultimately reducing therapeutic efficacy in solid tumors such as OS.

In conclusion, our study highlights the unaltered NKG2D-CAR T cell function under isolated hypoxia stress in OS models suggesting that additional TME-associated factors should be considered.

## Supplementary Information

Below is the link to the electronic supplementary material.

Supplementary file 1 NKG2D ligand expression in vitro and ex vivo in OS models. Representative histograms of NKG2DL expression in Saos-2 and U-2 OS under hypoxia conditions 48 h after seeding. Red—expression on normoxia; purple—expression on hypoxia; gray—negative control

Supplementary file 2 In vitro antitumor activity of NKG2D-CAR T cells under hypoxia at 1:5 effector/target ratio. Luciferase-expressing OS tumor cells are cocultured with NKG2D-CAR T cells at 1:5 different effector/target cells ratio in normoxic vs hypoxic conditions. **A** Live OS cells were determined by luminescence measurements after NKG2D-CAR T cell coculture. Data were normalized to OS cells without CAR T cells (OS cell control). **B** IFN-ɣ and TNF-α levels are quantified by ELISA in the cocultured supernatants. Data are represented as mean ± SD of experiments with CAR T cells from 3 different donors. All experiments were performed in duplicate. ***, *p* < 0.001 by two-way ANOVA with Tukey´s post hoc test

Supplementary file 3 NKG2DL expression on 143B-OS cells after NKG2D-CAR T coculture. **A** The expression of MICA, MICB, ULBP2/5/6 was measured by flow cytometry on live 143B-OS cells after 48 h of coculture with NKG2D-CAR T at ratio 1:1 in normoxia (red) and hypoxia (purple). **B** MICA levels were quantified by ELISA in the cocultured supernatants. Data represent the mean ± SD, *p < 0.05, **p < 0.01, ***p < 0.001, by Tukey’s test

Supplementary file 4 OS-spheroids formation under normoxic and hypoxic conditions. GFP-OS cells were seeded in low fixation U-bottom P-96 microplates. To allow spheroid formation, plates were centrifuged directly after seeding. Analysis is performed 48 h later. **A** GFP fluorescence signal of OS-spheroids, developed under normoxia and hypoxia conditions, was acquired by fluorescence microscopy (4x). **B** Spheroid size (area), form (circularity), and fluorescence intensity (integrated density) were quantified and compared under the conditions. Data shown are mean +SD of two independent experiments. All experiments were performed in triplicate. *, *p* < 0.05; **, *p* < 0.01; by two-way ANOVA with Sidak post hoc test

## Data Availability

The data that supports the findings of this study are available from the corresponding authors upon reasonable request.

## Abbreviations

AUC: Area under the curve
E/T: Effector-to-target
H: Height
ICs: Immune checkpoints
L: Length
MFI: Mean fluorescence intensity
MIC: MHC class I-related chain
NKG2DL: NKG2D ligands
NSG: NOD.Cg-Prkdc^scid Il2rg^tm1Wjl/SzJ
OS: Osteosarcoma
TME: Tumor microenvironment
ULBP: UL16-binding proteins
W: Width
