# Supplementary figures and images for: Unaltered NKG2D-CAR T cell function under hypoxia in osteosarcoma in vitro
Source: Cancer Immunol Immunother. 2026 Feb 12;75(3):75. doi: 10.1007/s00262-026-04319-w (PMC12901808; doi:10.1007/s00262-026-04319-w)

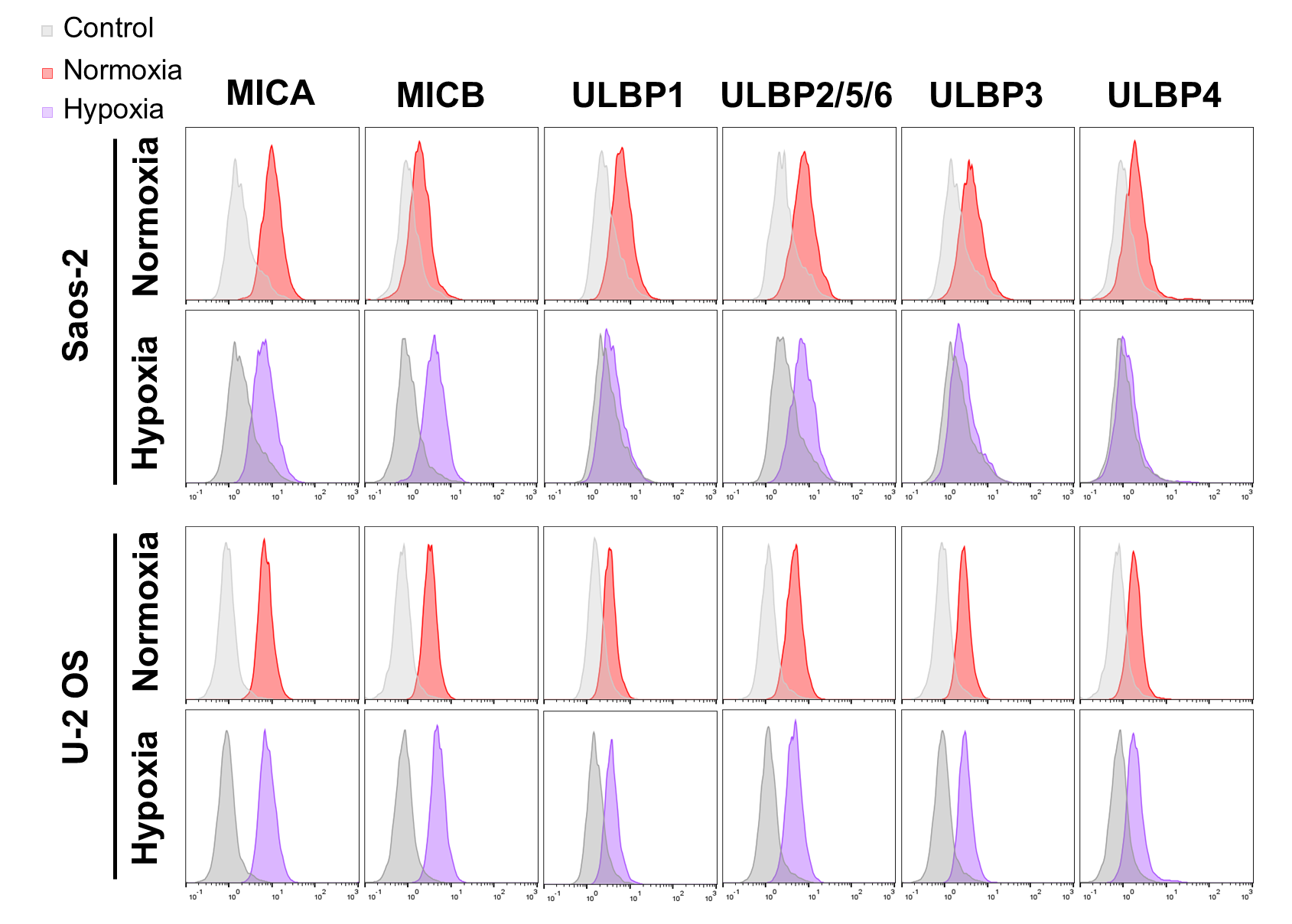

Supplement: Supplementary file 1 — NKG2D ligand expression in vitro and ex vivo in OS models. Representative histograms of NKG2DL expression in Saos-2 and U-2 OS under hypoxia conditions 48 h after seeding. Red—expression on normoxia; purple—expression on hypoxia; gray—negative control [file 262_2026_4319_MOESM1_ESM.tif]

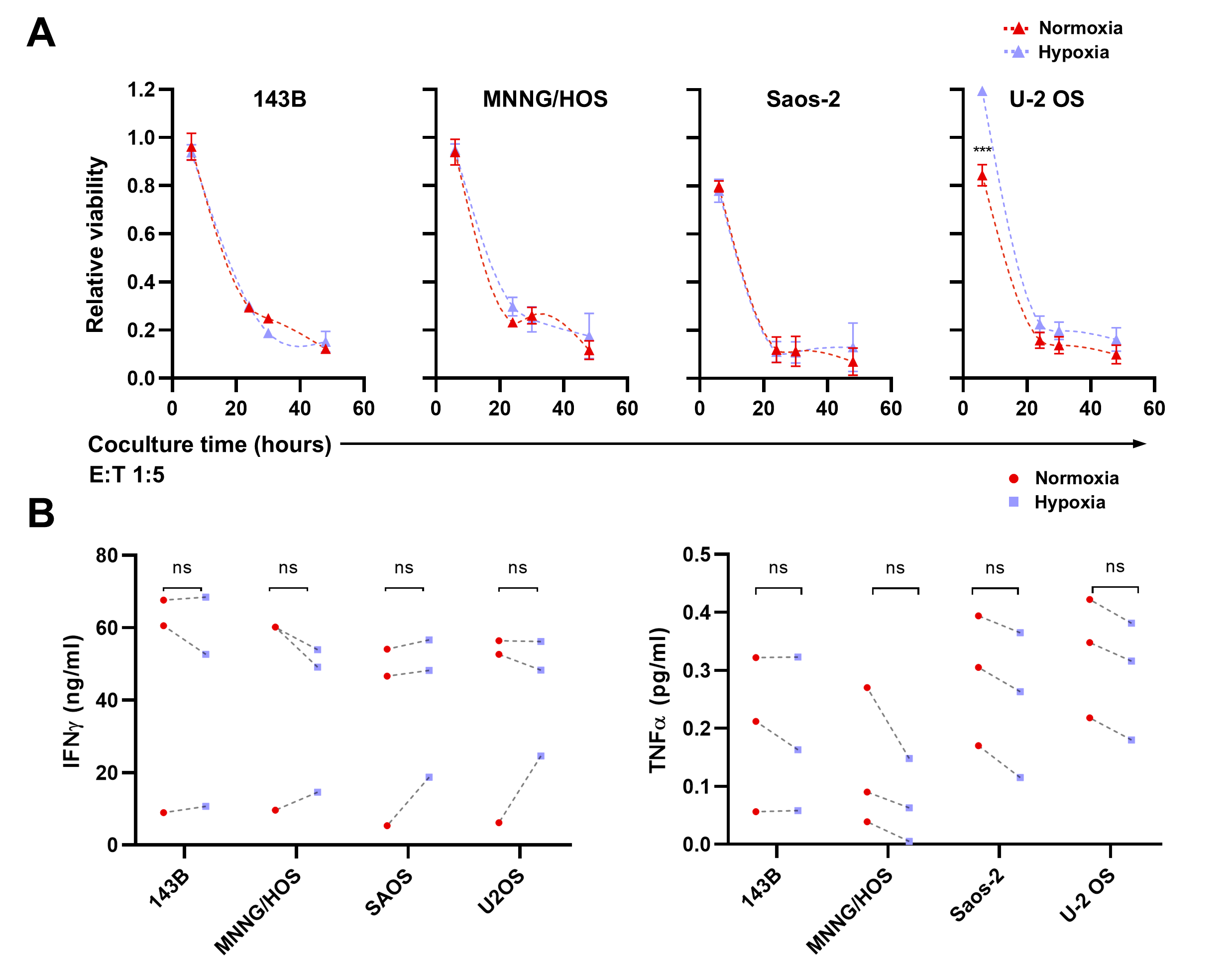

Supplement: Supplementary file 2 — In vitro antitumor activity of NKG2D-CAR T cells under hypoxia at 1:5 effector/target ratio. Luciferase-expressing OS tumor cells are cocultured with NKG2D-CAR T cells at 1:5 different effector/target cells ratio in normoxic vs hypoxic conditions. A Live OS cells were determined by luminescence measurements after NKG2D-CAR T cell coculture. Data were normalized to OS cells without CAR T cells (OS cell control). B IFN-ɣ and TNF-α levels are quantified by ELISA in the cocultured supernatants. Data are represented as mean ± SD of experiments with CAR T cells from 3 different donors. All experiments were performed in duplicate. ***, p < 0.001 by two-way ANOVA with Tukey´s post hoc test [file 262_2026_4319_MOESM2_ESM.tif]

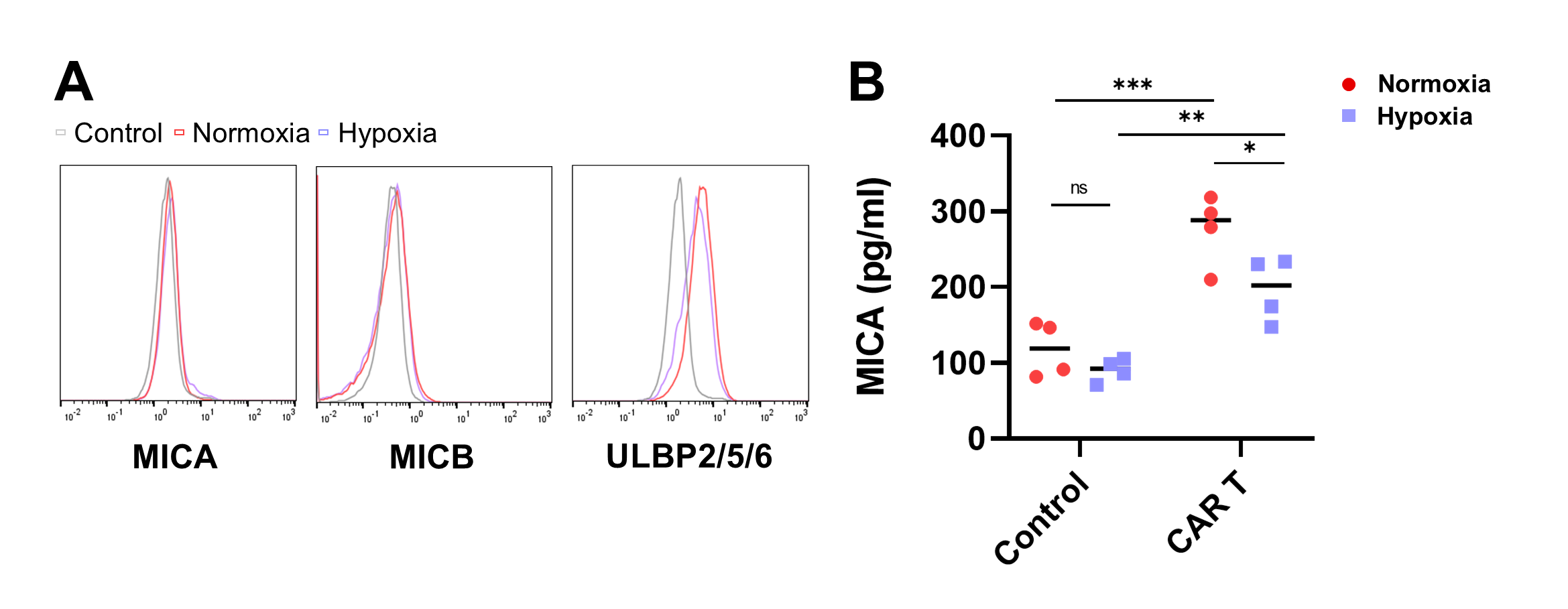

Supplement: Supplementary file 3 — NKG2DL expression on 143B-OS cells after NKG2D-CAR T coculture. A The expression of MICA, MICB, ULBP2/5/6 was measured by flow cytometry on live 143B-OS cells after 48 h of coculture with NKG2D-CAR T at ratio 1:1 in normoxia (red) and hypoxia (purple). B MICA levels were quantified by ELISA in the cocultured supernatants. Data represent the mean ± SD, *p < 0.05, **p < 0.01, ***p < 0.001, by Tukey’s test [file 262_2026_4319_MOESM3_ESM.tif]

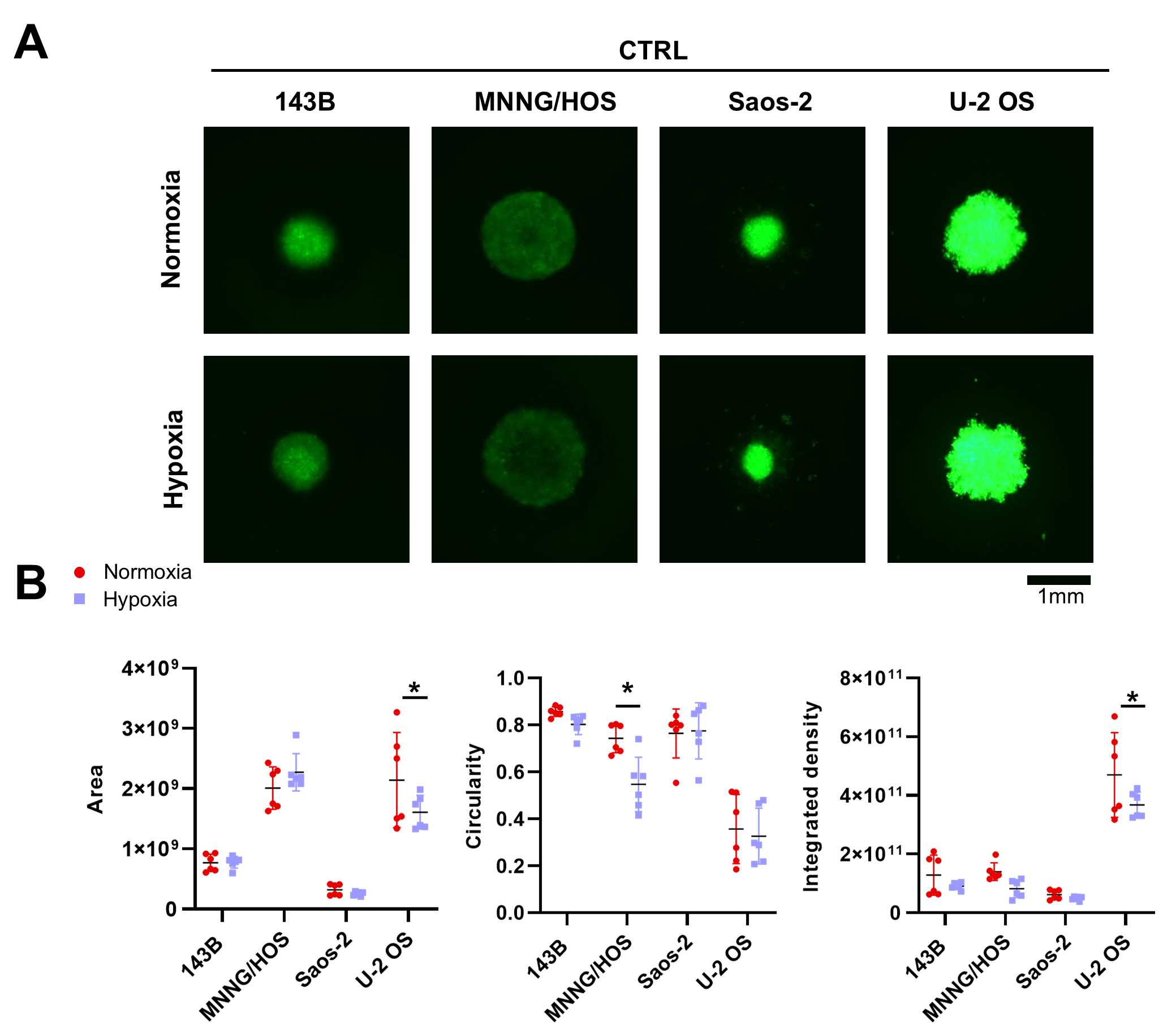

Supplement: Supplementary file 4 — OS-spheroids formation under normoxic and hypoxic conditions. GFP-OS cells were seeded in low fixation U-bottom P-96 microplates. To allow spheroid formation, plates were centrifuged directly after seeding. Analysis is performed 48 h later. A GFP fluorescence signal of OS-spheroids, developed under normoxia and hypoxia conditions, was acquired by fluorescence microscopy (4x). B Spheroid size (area), form (circularity), and fluorescence intensity (integrated density) were quantified and compared under the conditions. Data shown are mean +SD of two independent experiments. All experiments were performed in triplicate. *, p < 0.05; **, p < 0.01; by two-way ANOVA with Sidak post hoc test [file 262_2026_4319_MOESM4_ESM.tif]
